# Spectrochemical differentiation of meningioma tumours based on attenuated total reflection Fourier-transform infrared (ATR-FTIR) spectroscopy

**DOI:** 10.1007/s00216-019-02332-w

**Published:** 2019-12-21

**Authors:** Taha Lilo, Camilo L. M. Morais, Katherine M. Ashton, Ana Pardilho, Charles Davis, Timothy P. Dawson, Nihal Gurusinghe, Francis L. Martin

**Affiliations:** 1 Department of Neurosurgery, Royal Preston Hospital, Lancashire Teaching Hospitals NHS Trust, Preston, PR2 9HT UK; 2 School of Pharmacy and Biomedical Sciences, UCLan, Preston, PR1 2HE UK; 3 Department of Neuropathology, Royal Preston Hospital, Lancashire Teaching Hospitals NHS Trust, Preston, PR2 9HT UK

**Keywords:** Meningioma, Infrared spectroscopy, ATR-FTIR, Chemometrics

## Abstract

Meningiomas are the commonest types of tumours in the central nervous system (CNS). It is a benign type of tumour divided into three WHO grades (I, II and III) associated with tumour growth rate and likelihood of recurrence, where surgical outcomes and patient treatments are dependent on the meningioma grade and histological subtype. The development of alternative approaches based on attenuated total reflection Fourier-transform infrared (ATR-FTIR) spectroscopy could aid meningioma grade determination and its biospectrochemical profiling in an automated fashion. Herein, ATR-FTIR in combination with chemometric techniques is employed to distinguish grade I, grade II and grade I meningiomas that re-occurred. Ninety-nine patients were investigated in this study where their formalin-fixed paraffin-embedded (FFPE) brain tissue samples were analysed by ATR-FTIR spectroscopy. Subsequent classification was performed via principal component analysis plus linear discriminant analysis (PCA-LDA) and partial least squares plus discriminant analysis (PLS-DA). PLS-DA gave the best results where grade I and grade II meningiomas were discriminated with 79% accuracy, 80% sensitivity and 73% specificity, while grade I versus grade I recurrence and grade II versus grade I recurrence were discriminated with 94% accuracy (94% sensitivity and specificity) and 97% accuracy (97% sensitivity and 100% specificity), respectively. Several wavenumbers were identified as possible biomarkers towards tumour differentiation. The majority of these were associated with lipids, protein, DNA/RNA and carbohydrate alterations. These findings demonstrate the potential of ATR-FTIR spectroscopy towards meningioma grade discrimination as a fast, low-cost, non-destructive and sensitive tool for clinical settings.

**Electronic supplementary material:**

The online version of this article (10.1007/s00216-019-02332-w) contains supplementary material, which is available to authorized users.

**Graphical abstract:**
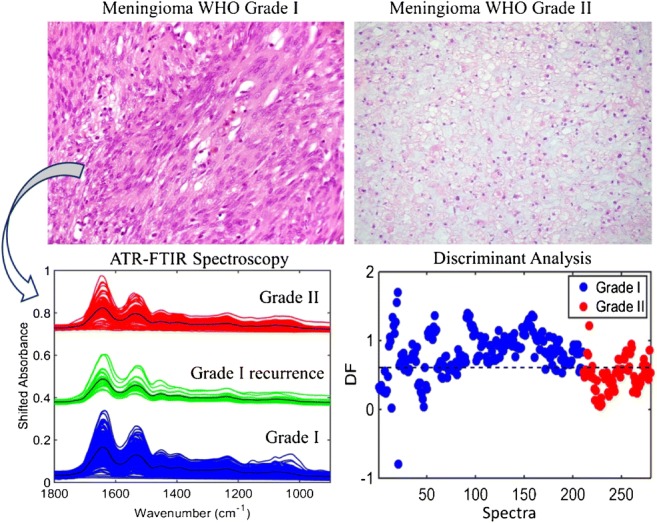
Attenuated total reflection Fourier-transform infrared (ATR-FTIR) spectroscopy was used to discriminate meningioma WHO grade I, grade II and grade I recurrence tumours.

## Introduction

Meningioma and glioma tumours constitute the majority of primary brain cancers [1]. Gliomas are a more aggressive and intrinsic type of tumour, which comprise neuroepithelial tumours originating from the glial or supporting cells of the central nervous system (CNS) [2]. Meningiomas are the commonest type of brain tumours, showing differentiation towards the meninges surrounding the brain and the spinal cord [3]. They are slow-growing extrinsic tumours with variable prognosis, occasionally growing to a very large size. The majority occur in a supratentorial location, while a few can arise in the posterior cranial fossa and, rarely, as extracranial meningiomas [4]. They often manifest as single or sporadic lesions, producing non-descript symptoms. The symptoms are variable in nature depending on the location and the size of the lesion. The most common presenting symptom is headache. However, symptoms may include any sensory and/or motor deficits and gait disturbance.

Multiple meningiomas are commonly associated with neurofibromatosis type II [5]. Meningiomas can be divided into WHO grade I (benign), grade II (atypical) and grade III (anaplastic) [3]. Grade I meningiomas are the commonest type of tumours, with slower growth and lower likelihood of recurrence, exhibiting histological patterns other than papillary, chordoid, clear cell or rhabdoid [6]; grade II meningiomas also have a slower growth but higher likelihood of recurrence, exhibiting 3 of the following parameters (macronuclei, spontaneous necrosis, hypercellularity, small cell formation, sheeting architecture), and have clear cell or chordoid cell types [6]; and grade III meningiomas are a very rare type of tumour with fast growing rate and much higher likelihood of recurrence, being cancerous aggressive, and histologically, they can resemble other tumours (melanomas, carcinomas and sarcomas) [6]. Accurate histological assessment is important since surgical outcomes and treatment are dependent on the meningioma grade and subtypes [5]. Moreover, the degree of surgical resection and Simpson grading have an important role also in patient outcome.

The pathogenesis and molecular genetics of meningioma are not very well understood; however, signalling pathways and growth factor alterations have been reported [6]. For example, cell cycle dysregulation and telomerase activation have been recognised as important steps in meningioma progression, where telomerase dynamics, cell cycle control and the mechanisms responsible for deoxyribonucleic acid damage control are highly entwined [6, 7]. Some WHO grade I meningiomas are linked to mutations of the *NF2* gene on chromosome 22 (location q12.2), which leads to the condition known as neurofibromatosis type II where benign tumour proliferates through the CNS [6]. *NF2* codes for the protein merlin that acts as a tumour suppressor in many different cell types. This protein interacts with the intermolecular amino-terminal domain and the carboxyl-terminal domain through phosphorylation that also controls the binding to its effector proteins [6]. In WHO grade I meningiomas, other proteins of 4.1 family members can also be downregulated, such as 4.1B (DAL-1) [6, 8]. Additionally, epidermal growth factor receptors (EGFRs) tend be overexpressed in grade I meningiomas, and the platelet-derived growth factor receptor beta (*PDGFRB*) gene is upregulated and overexpressed in this type of tumour [6]. On the other hand, WHO grade II meningiomas are characterised by chromosome mutations (mainly deletions) [6, 9], such as mutations in the 1p and 14q regions responsible for housing tumour suppressor genes, and further alterations in chromosome 1 [6]. Alterations in chromosome 10, including the *PTEN* gene located near the p23.3 region of chromosome 10, and other genetic non-*NF2* aberrations are associated with higher-grade meningiomas [6, 9]. *PTEN* is responsible for tumour suppression and production of the phosphatidylinositol-3,4,5-triphosphate 3-phosphate protein negatively that regulates the AKT/PKG pathway that has been linked to the pathogenesis and proliferation of meningiomas and other tumours [6, 9].

Infrared (IR) spectroscopy is a powerful technique to investigate biological materials [10]. The interaction of IR with the biochemical molecules that make up a tissue sample generates a spectrochemical fingerprint, allowing one to extract both quantitative and qualitative information. The IR signal, obtained by a change of the molecular dipole moment, reflects vibrational movements by the chemical bonds in the material, such as stretching and bending vibrations. This signal is specific for each type of sample and can be used to diagnose samples and to identify possible spectral biomarkers associated with tumour appearance and differentiation [11]. The signal within the fingerprint region (1800–900 cm^−1^) contains important spectrochemical signatures of key biomolecules, such as lipids (C=O symmetric stretching at 1750 cm^−1^, CH_2_ bending at 1470 cm^−1^), proteins (Amide I at 1650 cm^−1^, Amide II at 1550 cm^−1^, Amide III at 1260 cm^−1^), nucleic acids (asymmetric phosphate stretching at 1225 cm^−1^, symmetric phosphate stretching at 1080 cm^−1^), carbohydrates (CO–O–C symmetric stretching at 1155 cm^−1^), glycogen (C–O stretching at 1030 cm^−1^) and protein phosphorylation (970 cm^−1^) [10, 12, 13].

In order to obtain meaningful and reliable information, the IR spectra within the fingerprint region are processed through specific computational techniques, known as chemometrics. The spectral data initially undergo pre-processing techniques to correct the baseline and to remove possible physical variations not related to disease changes, and then chemometric models are built and validated, whereby possible spectral biomarkers as well as sensitivity and specificity metrics can be obtained [14]. Multivariate classification models, such as principal component analysis plus linear discriminant analysis (PCA-LDA) [15] and partial least squares plus discriminant analysis (PLS-DA) [16], are commonly employed to process IR spectral data, since these techniques allow to extract relevant spectral features associated with tumour differentiation and also to classify the samples into groups in a predictive fashion.

Gliomas comprise neuroepithelial tumours differentiating towards the glial or supporting cells of the CNS [2]. They are broadly classified into glioblastomas, astrocytomas, oligodendrogliomas, ependyomas and glioneuronal tumours [2]. Gliomas have been widely investigated using IR spectroscopy [16–23], while meningiomas have attracted relatively little attention [1, 24]. Meningiomas represent 20% to 35% of all primary intracranial tumours [4], and determining their WHO grade is essential to define appropriate treatment pathways. Herein, IR spectroscopy was applied to distinguish WHO grade I, II and I meningioma tumours that recurred.

## Materials and methods

### Samples and spectral acquisition

Ninety-nine 10-μm-thick formalin-fixed paraffin-embedded (FFPE) brain tissue samples (70 WHO grade I meningiomas, 24 WHO grade II meningiomas and 5 WHO grade I meningiomas that re-occurred) were analysed by a Bruker Tensor 27 FTIR spectrometer with Helios ATR attachment (Bruker Optics Ltd., Coventry, UK). All samples were sourced from the Brain Tumour North West (BTNW) biobank (National Research Ethics Service’s ethics approval NRES14/EE/1270). All experiments were approved by the STEMH (Science, Technology, Engineering, Medicine and Health) ethics committee at the University of Central Lancashire (STEMH 917). The H&E images for all samples are available upon reasonable request to the BTWN biobank; the sample details are depicted in Electronic Supplementary Material (ESM) Table S1. The sampling area, defined by an internal reflective element (diamond crystal), was approximately 250 μm × 250 μm. Samples were placed onto aluminium-covered slides [25], which, in turn, were affixed onto a moving platform with the sample facing up. De-parrafinisation was performed prior to commencing measurements using local protocols using xylene and ethanol [10]. Moving the platform upward allowed the specimen to contact the diamond crystal for spectral acquisition. Spectral resolution was 8 cm^−1^, over the range between 4000 and 400 cm^−1^, with 32 co-addition scans. Ten spectra were collected per tissue sample in different random locations to minimise bias. After each sample, the ATR crystal was cleaned with distilled water and a new background spectrum was acquired to take into account ambient changes before the next sampling. The time to analyse each tissue sample was approximately 10 min.

### Data analysis

The spectral data analysis was performed within a MATLAB R2014b environment (MathWorks, Natick, USA) using the Classification Toolbox for MATLAB [26]. The biofingerprint spectra (1800–900 cm^−1^) were pre-processed by Savitzky-Golay 2^nd^ derivative (window of 7 points, 2^nd^-order polynomial fit) and vector normalisation, a common pre-process employed in biological-derived spectral data for correcting random noise and baseline distortions and to improve the signal-to-noise ratio [10, 14]. An outlier test was performed using Hotelling’s *T*^2^ versus *Q* residual test [14], and no spectral outlier was observed in the dataset (see ESM Fig. S3). Thereafter, the samples for grade I and grade II meningiomas were divided into training (70% of samples) and validation (30%) sets using the Kennard-Stone uniform sample selection algorithm [27]. Cross-validated PCA-LDA and PLS-DA were built using venetian blinds cross-validation with 10 data splits.

PCA-LDA is a supervised discriminant analysis algorithm based on a principal component analysis (PCA) model followed by a linear discriminant analysis (LDA) classifier [15]. Initially, the pre-processed spectral data is reduced by PCA to a small number of principal components (PCs) accounting for the majority of the data explained variance [28]. Each PC is composed of scores and loadings: the first representing the variance on sample direction, thus being used to assess similarities/dissimilarities between samples, and the latter representing the variance on wavenumber direction, being used to find important spectral biomarkers. Then, a LDA model is built using the PCA scores, where the samples are assigned to classes based on a Mahalanobis distance calculation [15]. PLS-DA is another very popular supervised discriminant analysis technique that combines feature extraction and classification in a single routine [16]. In PLS-DA, a partial least squares (PLS) model is applied to the data reducing the pre-processed spectral data to a few numbers of latent variables (LVs); however, different from PCA-LDA, the input class labels for the training samples (e.g. + 1 or − 1) are used during this process, since PLS maximises the co-variation between the spectral information and the sample category. The samples are assigned to classes based on a straight line that divides the classes’ spaces [16].

### Model evaluation

The classification models were validated through the calculation of the accuracy, sensitivity and specificity in the validation set. Accuracy represents the total number of samples correctly classified considering true and false negatives, sensitivity represents the proportion of positive samples that are correctly classified and specificity represents the proportion of negative samples that are correctly classified [29]. These metrics are calculated as follows:

1 $$ \mathrm{Accuracy}\ \left(\%\right)=\frac{\mathrm{TP}+\mathrm{TN}}{\mathrm{TP}+\mathrm{FP}+\mathrm{TN}+\mathrm{FN}}\times 100 $$

2 $$ \mathrm{Sensitivity}\ \left(\%\right)=\frac{\mathrm{TP}}{\mathrm{TP}+\mathrm{FN}}\times 100 $$

3 $$ \mathrm{Specificity}\ \left(\%\right)=\frac{\mathrm{TN}}{\mathrm{TN}+\mathrm{FP}}\times 100 $$

where TP stands for true positives, TN for true negatives, FP for false positives and FN for false negatives.

## Results

**Fig. 1:**
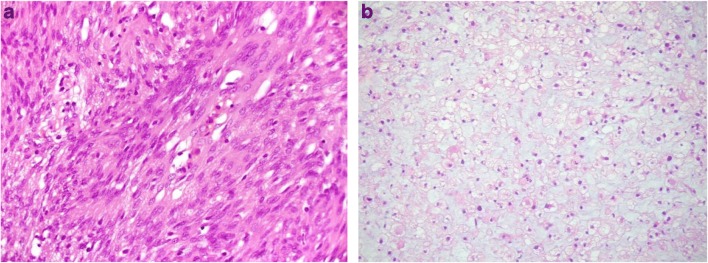
H&E slides. (**a**) WHO grade I meningioma (transitional meningioma). (**b**) WHO grade II meningioma (clear cell)

**Fig. 2:**
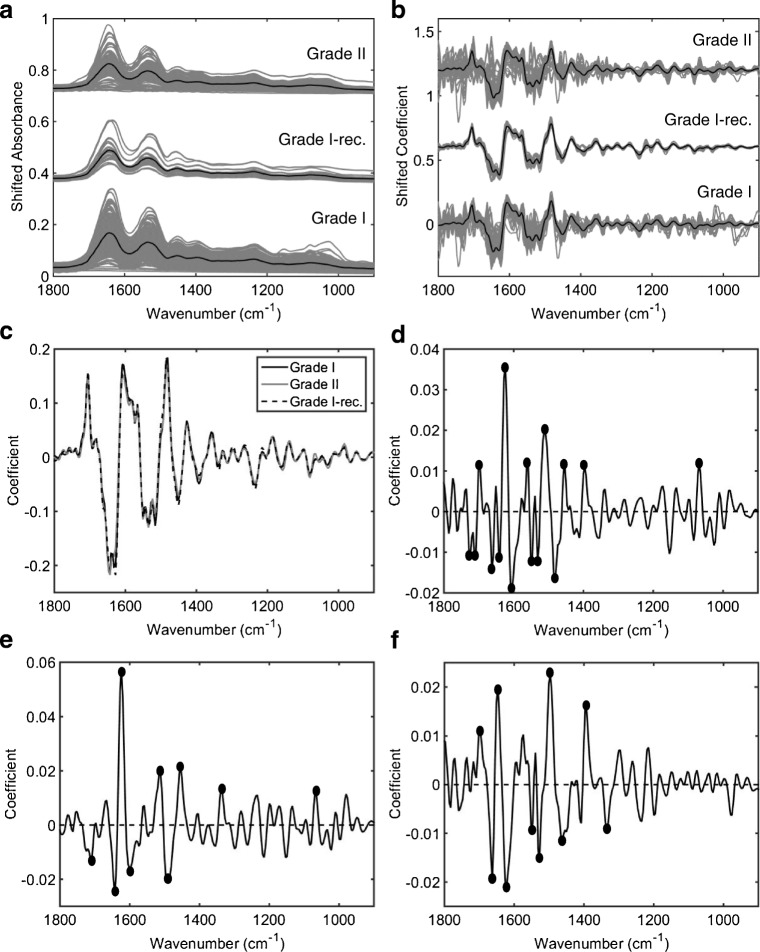
Infrared spectra for meningioma tumour samples (grade I, grade I recurrence and grade II). (**a**) Raw spectra and (**b**) pre-processed spectra (Savitzky-Golay 2^nd^ derivative and vector normalisation), where black line represents mean spectrum. (**c**) Mean spectrum for each class overlaid. (**d**) Difference-between-mean (DBM) spectrum for grade II (+) vs. grade I (−) meningiomas. (**e**) DBM spectrum for grade II (+) vs. grade I recurrence (−) meningiomas. (**f**) DBM spectrum for grade I recurrence (+) vs. grade I (−) meningiomas, where solid dots represent spectral wavenumbers with absolute coefficients > 0.01

This study is composed of 99 patients separated into 3 groups: grade I meningiomas (*n* = 70, 700 spectra), grade II meningiomas (*n* = 24, 240 spectra) and grade I meningiomas that re-occurred (*n* = 5, 50 spectra) (see ESM Table S1). Sample groups were pre-defined based on histopathologic evidence before spectral acquisition. Figure 1 shows an example of H&E slide for WHO grade I and grade II meningiomas. The raw and pre-processed (Savitzky-Golay 2^nd^ derivative and vector normalisation) IR spectra for each sample class are shown in Fig. 2 a–c and in Figs. S1 and S2 (see ESM). Grade I and grade II meningiomas exhibit higher levels of variability in comparison with grade I recurrence most likely due to the smaller number of grade I recurrence spectra (Fig. 2b). The difference-between-mean (DBM) spectrum for grade II (+ coefficients) and grade I (− coefficients) meningiomas is shown in Fig. 2 d, where 15 spectral markers were found with absolute coefficient intensity > 0.01: 1725 cm^−1^ (lower coefficient in grade II, C=O stretching in fatty acids), 1708 cm^−1^ (lower coefficient in grade II, C=O stretching in thymine), 1698 cm^−1^ (higher coefficient in grade II, C_2_=O stretching in guanine), 1663 cm^−1^ (lower coefficient in grade II, C=O stretching in cytosine), 1639 cm^−1^ (lower coefficient in grade II, Amide I), 1624 cm^−1^ (higher coefficient in grade II, base carbonyl stretching and ring breathing mode in nucleic acids), 1604 cm^−1^ (lower coefficient in grade II, adenine vibration in DNA), 1562 cm^−1^ (higher coefficient in grade II, ring base), 1550 cm^−1^ (lower coefficient in grade II, Amide II), 1530 cm^−1^ (lower coefficient in grade II, C=N and/or C=C stretching), 1512 cm^−1^ (higher coefficient in grade II, C–H in-plane bending), 1481 cm^−1^ (lower coefficient in grade II, Amide II), 1454 cm^−1^ (higher coefficient in grade II, asymmetric methyl deformation), 1396 cm^−1^ (higher coefficient in grade II, symmetric CH_3_ bending of the methyl groups of proteins) and 1068 cm^−1^ (higher coefficient in grade II, C–O stretching in ribose) [12]. Nine spectral markers with absolute coefficients > 0.01 were found in the DBM spectrum for grade II (+ coefficients) versus grade I recurrence (− coefficients) (Fig. 2e): 1708 cm^−1^ (lower coefficient in grade II, C=O stretching in thymine), 1643 cm^−1^ (lower coefficient in grade II, Amide I), 1624 cm^−1^ (higher coefficient in grade II, base carbonyl stretching and ring breathing mode in nucleic acids), 1600 cm^−1^ (lower coefficient in grade II, C=O stretching in lipids), 1512 cm^−1^ (higher coefficient in grade II, C–H in-plane bending), 1490 cm^−1^ (lower coefficient in grade II, C=C and/or in-plane C–H bending), 1454 cm^−1^ (higher coefficient in grade II, asymmetric methyl deformation), 1339 cm^−1^ (higher coefficient in grade II, collagen) and 1068 cm^−1^ (higher coefficient in grade II, C–O stretching in ribose) [12]. The DBM spectrum for grade I recurrence (+ coefficients) versus grade I (− coefficients) meningiomas (Fig. 1f) indicates 10 spectral markers with absolute coefficients > 0.01: 1698 cm^−1^ (higher coefficient in grade I recurrence, C_2_=O stretching in guanine), 1663 cm^−1^ (lower coefficient in grade I recurrence, C=O stretching in cytosine), 1647 cm^−1^ (higher coefficient in grade I recurrence, Amide I), 1624 cm^−1^ (lower coefficient in grade I recurrence, base carbonyl stretching and ring breathing mode in nucleic acids), 1550 cm^−1^ (lower coefficient in grade I recurrence, Amide II), 1527 cm^−1^ (lower coefficient in grade I recurrence, C=N and/or C=C stretching), 1496 cm^−1^ (higher coefficient in grade I recurrence, C=C stretching and/or C–H bending), 1460 cm^−1^ (lower coefficient in grade I recurrence, asymmetric CH_3_ bending in collagen), 1393 cm^−1^ (higher coefficient in grade I recurrence, symmetric CH_3_ bending in proteins) and 1335 cm^−1^ (lower coefficient in grade I recurrence, CH ring deformation in polysaccharides or pectin) [12].

The spectral data underwent chemometric analysis by means of PCA-LDA, as a first discriminant attempt, and then by PLS-DA as a final and best discriminant model. The following comparisons were investigated: (1) grade I versus grade II meningioma, (2) grade I versus grade I meningiomas that re-occurred and (3) grade II versus grade I meningiomas that re-occurred.

### Grade I versus grade II meningiomas

**Fig. 3:**
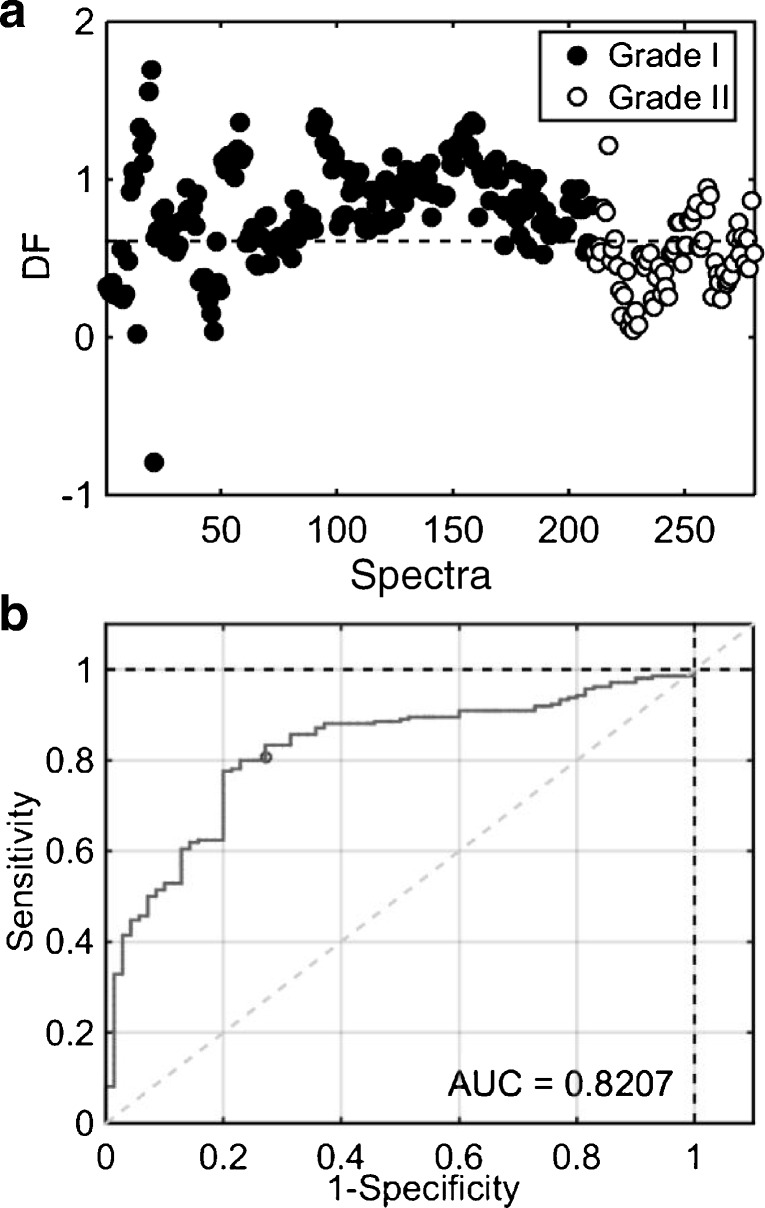
PLS-DA results to distinguish grade I vs. grade II meningiomas. (**a**) Discriminant function (DF) plot for samples’ spectra. (**b**) Receiver operating characteristic (ROC) curve, where AUC stands for area under the curve

The pre-processed data were initially separated into two subsets: training (70% of the samples) and validation (30% of the samples) using the KS algorithm. The training set was used for model construction while the validation set for final model evaluation. PCA-LDA was applied to the spectral data using 10 PCs (98% explained variance, see ESM Fig. S5), where training and validation accuracies were estimated at 89% and 71%, respectively (Table 1). Despite having reasonable accuracies and sensitivity (89% in the validation set), the specificity in validation was 20%, indicating that many grade I meningiomas were predicted as grade II. PLS-DA was then applied to the spectral data as a most powerful alternative for class differentiation. PLS-DA model was built with 11 LVs (93% spectral explained variance, see ESM Fig. S5), generating accuracies of 97% and 79% in the training and validation sets, respectively (Table 1). The sensitivity and specificity in the validation set were equal to 80% and 73%, respectively, with an area under the curve (AUC) value equal to 0.82, which indicates a good classification model. The PLS-DA discriminant function (DF) graph and receiver operating characteristic (ROC) curve to discriminate grade I and grade II meningiomas are depicted in Fig. 3. PLS-DA coefficients (see ESM Fig. S6) were used to extract biomarker information through an automatic peak detection algorithm that sought for the 8 most relevant peaks representing the wavenumbers with highest absolute coefficients (Table 2). PCA-LDA and PLS-DA model residuals are shown in ESM Fig. S4a and b.

**Table 1:** Quality metrics for PCA-LDA and PLS-DA models to distinguish grade I vs. grade II samples

Algorithm	Dataset	Accuracy (%)	Sensitivity (%)	Specificity (%)
PCA-LDA	Training	89	98	62
	Validation	71	89	20
PLS-DA	Training	97	96	99
	Validation	79	80	73

**Table 2:** Spectral markers identified by PLS-DA in order to discriminate grade I and grade II meningiomas

Wavenumber (cm^−1^)	Tentative assignment	Relative intensity^a^	*P* value
1651	Amide I	↓	0.035 (*)
1593	NH_2_ adenine	↓	< 10^−7^ (**)
1546	Amide II	↑	0.637
1500	*δ*(CH) in-plane	↑	< 10^−4^ (**)
1454	*δ*(CH_3_) asymmetric	↓	< 10^−13^ (**)
1377	*v*(C–O)	↓	0.030 (*)
1227	*v*_as_(PO_2_^−^)	↓	0.051
1122	*v*(C–O) in carbohydrates	↑	0.014 (*)

*P* value was calculated by an ANOVA test

*δ* bending, *v* stretching, *v*_as_ asymmetric stretching

*P value < 0.05 considered statistically significant; **P value < 0.001 considered statistically highly significant

^a^↑ = higher intensity in grade II meningioma; ↓ = lower intensity in grade II meningioma

### Grade I versus grade I meningiomas that re-occurred

**Fig. 4:**
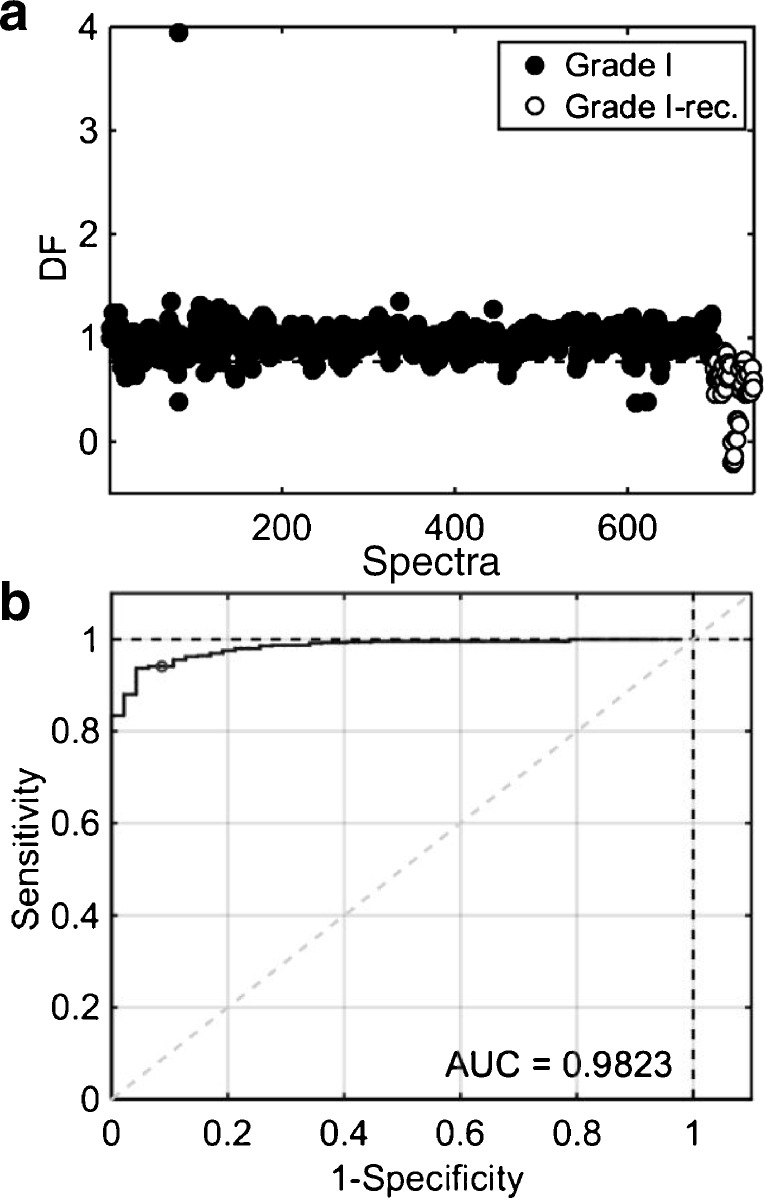
PLS-DA results to distinguish grade I vs. grade I recurrence meningiomas. (**a**) Discriminant function (DF) plot for samples’ spectra. (b) Receiver operating characteristic (ROC) curve, where AUC stands for area under the curve

Cross-validated PCA-LDA was applied to the spectral data using 17 PCs (97% explained variance) (ESM Fig. S7), where both training and validation accuracies were estimated at 95% (Table 3). Despite having excellent values of accuracy and sensitivity (99%), the specificity is again low in validation (32%), indicating that many grade I meningiomas were predicted as grade I that re-occurred. PLS-DA was applied to the spectral data with 17 LVs (95% spectral explained variance) (see ESM Fig. S7), generating accuracies of 96% and 94% in the training and validation sets, respectively (Table 3). The sensitivity and specificity in the validation were both equal to 94%, with an AUC value equal to 0.98, which indicates an almost perfect classification. The PLS-DA DF graph and ROC curve to discriminate grade I and grade I recurrence meningiomas are depicted in Fig. 4. The wavenumbers with highest absolute PLS-DA coefficients (see ESM Fig. S8) are depicted in Table 4. PCA-LDA and PLS-DA model residuals are shown in Fig. S4c and S4d (see ESM).

**Table 3:** Quality metrics for PCA-LDA and PLS-DA models to distinguish grade I vs. grade I recurrence samples

Algorithm	Dataset	Accuracy (%)	Sensitivity (%)	Specificity (%)
PCA-LDA	Training	95	99	34
	Validation	95	99	32
PLS-DA	Training	96	96	100
	Validation	94	94	94

**Table 4:** Spectral markers identified by PLS-DA in order to discriminate grade I and grade I recurrence meningiomas

Wavenumber (cm^−1^)	Tentative assignment	Relative intensity^a^	*P* value
1755	*v*(C=O) in lipids	↓	< 10^−3^ (**)
1693	Amide I (antiparallel β-sheet)	↑	< 10^−3^ (**)
1477	*δ*(CH_2_) in lipids	↓	0.229
1423	*δ*(CH_2_) in polysaccharides	↑	0.722
1400	*v*_s_(COO^−^) in amino acids (aspartate, glutamate)	↓	< 10^−5^ (**)
1369	*v*(C–N) in cytosine and guanine	↓	0.542
1346	*δ*(CH_2_) in collagen	↓	0.940
1246	*v*_as_(PO_2_^−^)	↑	< 10^−4^ (**)

*P* value was calculated by an ANOVA test

*δ* bending, *v* stretching, *v*_s_ symmetric stretching, *v*_as_ asymmetric stretching

**P value < 0.001 considered statistically highly significant

^a^↑ = higher intensity in grade I recurrence meningioma; ↓ = lower intensity in grade I recurrence meningioma

### Grade II versus grade I meningiomas that re-occurred

**Fig. 5:**
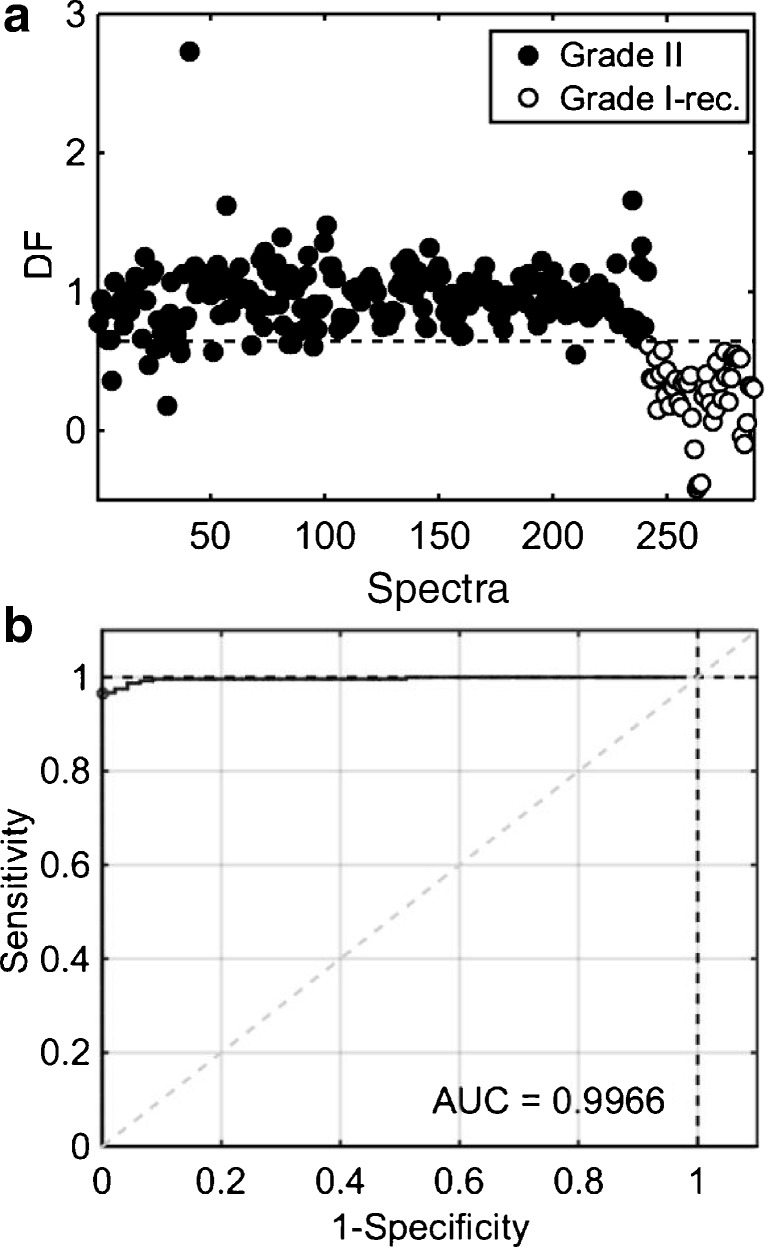
PLS-DA results to distinguish grade II vs. grade I recurrence meningiomas. (**a**) Discriminant function (DF) plot for samples’ spectra. (**b**) Receiver operating characteristic (ROC) curve, where AUC stands for area under the curve

Cross-validated PCA-LDA was applied to the spectral data using 12 PCs (96% explained variance) (see ESM Fig. S9), where both training and validation accuracies were estimated at 90% (Table 5). Once more, the specificity of PCA-LDA is highly affected (45%), despite having the good accuracies and sensitivity (99%). PLS-DA was applied to the spectral data with 13 LVs (95% spectral explained variance) (see ESM Fig. S9), generating accuracies of 99% and 97% in the training and validation sets, respectively (Table 5). The sensitivity and specificity in the validation were both equal to 97%, with an AUC value equal to 0.99, which indicates a close-to-perfect classification. The PLS-DA DF graph and ROC curve to discriminate grade II and grade I recurrence meningiomas are depicted in Fig. 5, where the wavenumbers with highest absolute PLS-DA coefficients are depicted in Table 6. PCA-LDA and PLS-DA model residuals are shown in Fig. S4e and S4f (see ESM).

**Table 5:** Quality metrics for PCA-LDA and PLS-DA models to distinguish grade II vs. grade I recurrence samples

Algorithm	Dataset	Accuracy (%)	Sensitivity (%)	Specificity (%)
PCA-LDA	Training	90	99	47
	Validation	90	98	45
PLS-DA	Training	99	98	100
	Validation	97	97	100

**Table 6:** Spectral markers identified by PLS-DA in order to discriminate grade II and grade I recurrence meningiomas

Wavenumber (cm^−1^)	Tentative assignment	Relative intensity^a^	*P* value
1639	Amide I	↓	0.579
1597	NH_2_ adenine	↑	0.001 (*)
1547	Amide II	↓	0.425
1523	*v*(C=N)	↑	0.018 (*)
1454	*δ*(CH_3_) asymmetric	↑	< 10^−3^ (**)
1265	*v*_as_(PO_2_^−^)	↑	< 10^−11^ (**)
1242	Amide III	↑	< 10^−5^ (**)
1122	*v*(C–O) in carbohydrates	↓	0.251

*P* value was calculated by an ANOVA test

*P value < 0.05 considered statistically significant; **P value < 0.001 considered statistically highly significant

^a^↑ = higher intensity in grade I recurrence meningioma; ↓ = lower intensity in grade I recurrence meningioma

## Discussion

Normal and tumour brain tissues have been previously discriminated using IR or Raman spectroscopy [1], where neoplastic tissues (meningioma, glioma and brain metastasis) were found to be statically significant from normal tissues using PCA-LDA as the multivariate spectral analysis technique. Hands et al. [30] reported serum diagnostic of brain tumours using ATR-FTIR spectroscopy with support vector machines (SVMs) with the sensitivity of 89.4% and specificity of 78.0% to distinguish cancerous from non-cancerous samples and the sensitivity of 82.1% and specificity of 75.0% to distinguish glioma from meningioma tissue. Bury et al. [31] reported the use of ATR-FTIR spectroscopy to analyse plasma samples in order to distinguish non-cancer from different cancerous brain samples. Normal and meningioma samples were differentiated with 87% accuracy using PCA-LDA and 95% accuracy using SVM, and meningioma samples were diagnosed among several groups of samples (normal, high-grade glioma, low-grade glioma and brain metastasis) with an accuracy of 63% using PCA-LDA and an accuracy of 100% using SVM.

Herein, WHO grade I and grade II meningiomas were discriminated with 79% accuracy in the validation set (80% sensitivity, 73% specificity, AUC = 0.82) using PLS-DA, a simpler and less susceptible method to overfitting than SVM, indicating a satisfactory clinical performance taking into consideration the complexity of the data obtained, as demonstrated by the patient demographics in Table S1 (see ESM) and the inherent spectrochemical complexity of tissue samples. Despite having high accuracies and sensitivities, the lower specificities of the PCA-LDA models indicate that these models are skewed towards the bigger class size, so the model is classifying the samples from this class more accurately (high sensitivity) than the samples from the smaller class (low specificity). The accuracy is influenced by the class size, so it tends to follow the sensitivity. The PLS-DA models, on the other hand, have a better consistency between sensitivity and specificity, thus indicating no overfitting. The statistically significant spectral markers were mainly associated with proteins (Amide I, Amide II), carbohydrates (*v*(C–O)) and DNA/RNA functional groups (NH_2_ adenine, *v*_as_(PO_2_^−^)) (Table 2). Proteins play an important role in the molecular pathways for meningiomas, where, for example, integrin exhibits different expression profiles within different grades of meningioma [32]. In addition, Amide I, Amide II and carbohydrate absorptions have been associated with differences between normal and meningioma tissues [31], and *δ*(CH), *δ*(CH_3_), *v*(C–O) and *v*_as_(PO_2_^−^) have been found to be related to spectral markers associated with brain tumours in general [30]. These findings indicate that IR spectroscopy allied with chemometrics could be used to aid clinical differentiation of grade I and grade II meningioma tumours in a non-destructive, fast and sensitive way.

“Grade I” and “grade I recurrence” were found to be clearly different, in which a discriminant performance of 94% accuracy (94% sensitivity and specificity) was obtained to distinguish both types of tumours. This indicates that one can assess the presence of recurrence in comparison with regular grade I tumours in an objective and automatic fashion by using IR spectroscopy and chemometrics. This is immensely important stratification information, which cannot be routinely derived or inferred from the histological examination of meningiomas lying within a WHO grade. The spectral markers associated with recurrence (Table 4) were mainly protein (Amide I), lipids, collagen and DNA/RNA changes (*v*_as_(PO_2_^−^)). DNA alterations, in special DNA methylation, are highly associated with meningioma progression, especially as a discriminant feature between *NF2*-mutated and non-*NF2*-mutated tumours [33]. By evaluating the spectral profile of all patients in grade I cohort, 12 patients (patients 2, 11, 16, 22, 26, 30, 34, 38, 50, 56, 57 and 69) were found to have these spectral markers following the same trend observed in grade I recurrence, in terms of relative intensity. This corresponds to 17% of grade I cohort, and these patients could be potential candidates to have grade I re-occurring in the future, once their spectral marker profiles are similar to the ones in grade I recurrence cohort. In this case, these patients could be followed closely in the clinical scenario to evaluate if the tumour will re-occur in the future. Since meningioma re-occurs with an average time of 10 years, this pilot study does not have this confirmative information for these patients, although the estimated recurrence rate of 17% is close to the usual meningioma grade I recurrence rate of 10%. This is just a hypothesis that needs further validation, but if this methodology is proved true, one could use this spectrochemical information to follow up patients with higher likelihood of recurrence and provide them with more specific treatments and closer attention, reducing existent costs associated with unknown recurrence odds.

Finally, grade II and grade I recurrence were discriminated based on their spectrochemical profile with an accuracy of 97% (97% sensitivity and 100% specificity) and the main spectral markers associated with recurrence (Table 6) were proteins (Amide I, Amide II and Amide III), carbohydrates (*v*(C–O)) and DNA/RNA alterations (NH_2_ adenine, *v*_as_(PO_2_^−^)), therefore indicating that these tumour types are very different. An important advantage of using ATR-FTIR spectroscopy is that due to its non-destructive nature [34], the same tissue section could theoretically be used for conventional histological analysis or other complementary techniques such as Raman microspectroscopy [35]. Moreover, the sensitivity and specificity for meningioma tumour detection towards clinical diagnosis might improve in future applications using FTIR microspectroscopy due to its relatively larger spatial resolution in comparison with ATR-FTIR spectroscopy, which enables the acquisition of richer spatially distributed spectrochemical information.

## Electronic supplementary material

ESM 1 (DOCX 890 kb)
