# Supplementary material for: Spectrochemical differentiation of meningioma tumours based on attenuated total reflection Fourier-transform infrared (ATR-FTIR) spectroscopy
Source: Anal Bioanal Chem. 2019 Dec 21;412(5):1077–86. doi: 10.1007/s00216-019-02332-w (PMC7007428; doi:10.1007/s00216-019-02332-w)
Supplement: Supplementary file 1 — (DOCX 890 kb) [file 216_2019_2332_MOESM1_ESM.docx]

**Analytical and Bioanalytical Chemistry**

**Electronic Supplementary Material**

**Spectrochemical differentiation of meningioma tumours based on attenuated total reflection Fourier-transform infrared (ATR-FTIR) spectroscopy**

Taha Lilo, Camilo L.M. Morais, Katherine M. Ashton, Ana Pardilho, Charles Davis,
Timothy P. Dawson, Nihal Gurusinghe, Francis L. Martin

**Table S1** Patients’ characteristics

| **Patient Number** | **WHO grade** | **Sex** | **Age** | **Histology** |
| --- | --- | --- | --- | --- |
| 1 | I | F | 67 | Fibrous |
| 2 | I | F | 63 | Transitional |
| 3 | I | M | 47 | Transitional |
| 4 | I | F | 70 | Transitional |
| 5 | I | F | 35 | Transitional |
| 6 | I | F | 54 | Fibrous |
| 7 | I | F | 53 | Fibrous |
| 8 | I | F | 43 | Transitional |
| 9 | I | F | 55 | Transitional |
| 10 | I | M | 70 | Transitional |
| 11 | I | F | 50 | Transitional |
| 12 | I | M | 55 | Transitional |
| 13 | I | F | 59 | Meningothelial |
| 14 | I | F | 65 | Meningothelial |
| 15 | I | M | 47 | Syncitial |
| 16 | I | M | 58 | Secretory type |
| 17 | I | M | 55 | Secretory type |
| 18 | I | M | 55 | Secretory type |
| 19 | I | F | 76 | Transitional |
| 20 | I | F | 50 | Meningothelial |
| 21 | I | F | 41 | Meningothelial |
| 22 | I | F | 41 | Transitional |
| 23 | I | M | 69 | Transitional |
| 24 | I | F | 48 | Fibrous |
| 25 | I | F | 52 | Fibrous |
| 26 | I | F | 54 | Secretory type |
| 27 | I | M | 88 | Fibrous |
| 28 | I | F | 86 | Fibrous |
| 29 | I | F | 76 | Fibrous |
| 30 | I | M | 76 | Syncitial |
| 31 | I | F | 59 | Fibrous |
| 32 | I | F | 52 | Fibrous |
| 33 | I | F | 36 | Fibrous |
| 34 | I | F | 50 | Fibrous |
| 35 | I | F | 73 | Syncitial |
| 36 | I | F | 58 | Transitional |
| 37 | I | M | 60 | Transitional |
| 38 | I | M | 55 | Transitional |
| 39 | I | F | 55 | Secretory type |
| 40 | I | F | 60 | Transitional |
| 41 | I | F | 76 | Transitional |
| 42 | I | M | 50 | Microcystic |
| 43 | I | F | 54 | Secretory type |
| 44 | I | F | 54 | Secretory type |
| 45 | I | M | 61 | Secretory type |
| 46 | I | F | 48 | Transitional |
| 47 | I | F | 77 | Secretory type |
| 48 | I | F | 42 | Fibrous |
| 49 | I | F | 68 | Transitional |
| 50 | I | F | 65 | Transitional |
| 51 | I | F | 81 | Meningothelial |
| 52 | I | F | 54 | Secretory type |
| 53 | I | M | 59 | Syncitial |
| 54 | I | F | 72 | Fibrous |
| 55 | I | F | 44 | Angiomatous |
| 56 | I | F | 64 | Psammomatous |
| 57 | I | F | 66 | Transitional |
| 58 | I | F | 51 | Transitional |
| 59 | I | F | 50 | Meningothelial |
| 60 | I | F | 58 | Meningothelial |
| 61 | I | F | 47 | Secretory type |
| 62 | I | F | 61 | Fibrous |
| 63 | I | F | 53 | Fibrous |
| 64 | I | F | 52 | Secretory type |
| 65 | I | F | 51 | Secretory type |
| 66 | I | F | 57 | Meningothelial |
| 67 | I | F | 58 | Fibrous |
| 68 | I | M | 39 | Angiomatous |
| 69 | I | F | 43 | Fibrous |
| 70 | I | F | 57 | Fibrous |
| 71 | II | F | 67 | Atypical |
| 72 | II | F | 67 | Atypical |
| 73 | II | M | 62 | Chordoid |
| 74 | II | M | 62 | Atypical |
| 75 | II | M | 57 | Atypical |
| 76 | II | F | 76 | Atypical |
| 77 | II | F | 66 | Chordoid |
| 78 | II | F | 53 | Chordoid |
| 79 | II | F | 58 | Atypical |
| 80 | II | M | 40 | Chordoid |
| 81 | II | M | 80 | Atypical |
| 82 | II | F | 44 | Atypical |
| 83 | II | F | 44 | Atypical |
| 84 | II | F | 60 | Atypical |
| 85 | II | F | 60 | Atypical |
| 86 | II | M | 66 | Atypical |
| 87 | II | F | 57 | Chordoid |
| 88 | II | F | 92 | Atypical |
| 89 | II | F | 79 | Atypical |
| 90 | II | M | 75 | Atypical |
| 91 | II | F | 75 | Atypical |
| 92 | II | F | 62 | Atypical |
| 93 | II | F | 62 | Atypical |
| 94 | II | M | 61 | Atypical |
| 95 | I-recurrence | M | 69 | Transitional |
| 96 | I-recurrence | F | 50 | Transitional |
| 97 | I-recurrence | M | 55 | Transitional |
| 98 | I-recurrence | F | 59 | Meningothelial |
| 99 | I-recurrence | F | 65 | Meningothelial |

**Fig. S1** Raw IR spectra for meningiomas WHO Grade I, II and I-recurrence


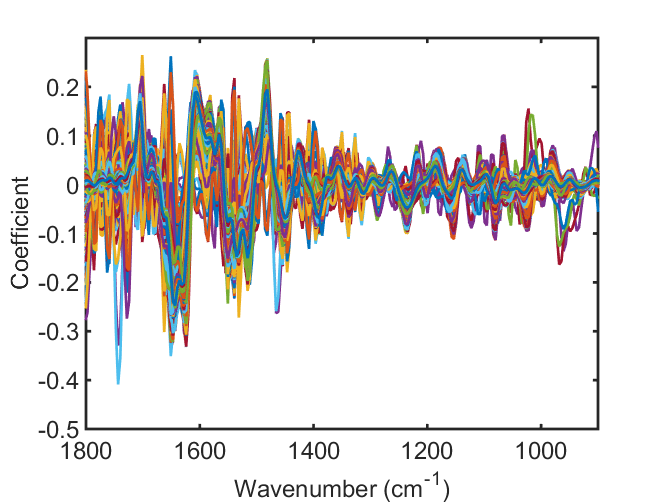


**Fig. S2** Pre-processed IR spectra (Savitzky-Golay 2^nd^ derivative and vector normalisation) for meningiomas WHO Grade I, II and I-recurrence

**Fig. S3** (a) PCA scores on PC1 *versus* PC2 for the pre-processed spectral data in the fingerprint region (Savitzky-Golay 2^nd^ derivative [window of 7 points, 2^nd^ order polynomial fit] and vector normalisation); (b) Hotelling’s T^2^ *versus* Q residuals chart (6 PCs, 85.57% explained variance). Grade I-rec. stands for WHO Grade I samples that reoccurred

**Fig. S4** Sum of squared residuals (SSR) for (a) PCA-LDA and (b) PLS-DA models to distinguish Grade I *vs.* Grade II meningiomas; (c) PCA-LDA and (d) PLS-DA models to distinguish Grade I *vs.* Grade I meningiomas that re-occurred; (e) and (f) PCA-LDA and PLS-DA models to distinguish Grade II *vs.* Grade I meningiomas that re-occurred

**Fig. S5** Cross-validation error for PCA-LDA and PLS-DA models to distinguish meningiomas grade I *vs.* grade II samples

**Fig. S6** PLS-DA coefficients to distinguish meningiomas grade I *vs.* grade II samples

**Fig. S7** Cross-validation error for PCA-LDA and PLS-DA models to distinguish meningiomas grade I *vs.* grade I recurrence samples

**Fig. S8** PLS-DA coefficients to distinguish meningiomas grade I *vs.* grade I recurrence samples

**Fig. S9** Cross-validation error for PCA-LDA and PLS-DA models to distinguish meningiomas grade II *vs.* grade I recurrence samples
